# Neonatal Herpes Simplex Virus Keratitis: Bitter Lessons from a Case Report

**DOI:** 10.1002/ccr3.72894

**Published:** 2026-06-15

**Authors:** Mohammad Saeid Sasan, Amin Saeidinia

**Affiliations:** ^1^ Faculty of Medicine Mashhad University of Medical Sciences Mashhad Iran; ^2^ Booali Research Institute, Pharmacological Research Center Mashhad University of Medical Sciences Mashhad Iran

**Keywords:** acyclovir, case report, encephalitis, keratitis, neonatal herpes

## Abstract

Neonatal herpes simplex virus (HSV) infection is an uncommon but potentially devastating condition. Ocular involvement, particularly herpetic keratitis, can cause significant morbidity if not promptly recognized and treated. We present a case of isolated HSV‐1 keratitis in a 25‐day‐old neonate successfully managed with intravenous acyclovir; however, the patient subsequently developed HSV encephalitis. This case underscores the importance of considering HSV infection in neonates presenting with ocular symptoms, even in the absence of systemic manifestations, and highlights the risk of late‐onset complications.

## Key Clinical Message

Neonatal HSV, even with isolated keratitis, requires high suspicion. All suspected cases need LP, neuroimaging, dilated eye exam, and blood PCR. After IV acyclovir, all three disease forms (SEM, CNS, disseminated) should receive 6 months of oral suppressive acyclovir. Vigilance for late encephalitis remains essential.

**Abbreviations**
CNS: central nervous system
CRP: C‐reactive protein
ESR: erythrocyte sedimentation rate
HSV: herpes simplex virus
MRI: magnetic resonance imaging
PCR: Polymerase chain reaction
SEM: skin eyes, and mouth

## 1 Introduction

Neonatal herpes simplex virus (HSV) infection remains a significant cause of neonatal morbidity and mortality, occurring in approximately 1 in 3000 to 1 in 10,000 live births [1]. Both HSV‐1 and HSV‐2 can cause neonatal infections, with HSV‐2 traditionally more associated with genital infections and HSV‐1 increasingly recognized in neonatal cases [1]. Neonatal HSV infection typically manifests as one of three clinical syndromes: disseminated disease, central nervous system (CNS) disease, or skin, eyes, and mouth (SEM) disease [2]. Ocular involvement may be part of systemic disease or, more rarely, an isolated presentation. Herpetic keratitis, characterized by corneal epithelial ulceration, stromal inflammation, and sometimes scarring, represents a critical ophthalmologic emergency in neonates due to its potential for rapid progression and vision loss [3, 4]. Herein, we report a case of isolated HSV‐1 keratitis in a neonate, successfully treated initially but complicated by HSV encephalitis weeks later, underscoring the importance of early diagnosis, aggressive management, and vigilant follow‐up.

## 2 Case History/Examination

A full‐term female neonate was born at 38 weeks gestation via an uncomplicated spontaneous vaginal delivery, with a birth weight of 2800 g and Apgar scores of 8 and 9 at 1 and 5 min, respectively. Maternal HSV serology was not performed because the mother denied any history of herpetic infection and had no active lesions during pregnancy. At the time of delivery, there were no visible herpetic lesions on the mother, and membrane rupture was spontaneous without prolonged duration (less than 6 h prior to delivery). The mother denied any history of herpes simplex infection or other sexually transmitted infections during pregnancy, and prenatal screening was unremarkable.

At 25 days of age, the infant presented with a three‐day history of left eye redness, purulent discharge, and increased irritability. The mother also noted photophobia and decreased willingness to open the affected eye. On clinical examination, marked conjunctival injection and palpebral edema were evident, accompanied by mucopurulent discharge. The cornea appeared opaque on gross inspection. Vesicular skin lesions were also observed on the left eyelid and surrounding periorbital area, which had been present since birth but unreported until this visit.

The infant was afebrile and hemodynamically stable. Neurological examination was within normal limits, with no evidence of seizures, focal deficits, or systemic signs such as respiratory distress or hepatosplenomegaly.

## 3 Differential Diagnosis, Investigations and Treatment

Initial diagnostic workup included a chest X‐ray that showed normal cardiothoracic ratio, clear lung fields, and no skeletal abnormalities. Laboratory tests revealed mild anemia (hemoglobin 10.2 g/dL) and thrombocytosis (platelet count 520,000/μL), while inflammatory markers including C‐reactive protein (CRP) and erythrocyte sedimentation rate (ESR) were within normal limits. Blood glucose, renal and liver function tests, and electrolytes were unremarkable.

Microbiological cultures and Gram stains of the eye discharge were negative for bacterial pathogens. Ophthalmological examination with fluorescein staining demonstrated a large geographic epithelial ulcer with irregular margins and underlying stromal haze, strongly suggestive of herpetic keratitis. Polymerase chain reaction (PCR) testing of conjunctival swabs and vesicular skin lesions confirmed the presence of HSV type 1 DNA. In all neonates with suspected HSV disease, a whole blood or plasma sample should also be tested for HSV using PCR [5].

Due to absence of neurological signs (normal neurological examination, no seizures, no lethargy, normal feeding), the isolated ocular presentation without systemic illness, and parental anxiety regarding the procedure, lumbar puncture was deferred. We acknowledge that current AAP/IDSA guidelines recommend lumbar puncture in all neonates with suspected HSV infection, even without neurological signs, because CNS involvement can be subclinical. Specifically, all neonates with suspected HSV, even those who appear to have isolated SEM disease, should undergo lumbar puncture because clinical findings may be absent early in the course of CNS disease [5]. In retrospect, lumbar puncture should have been performed [5]. The clinical picture was consistent with isolated neonatal HSV‐1 keratitis.

Furthermore, all infants with neonatal HSV disease, regardless of disease classification, should have neuroimaging performed (e.g., MRI or CT) and a dilated eye examination in addition to conjunctival HSV cultures to evaluate for eye involvement [5]. The infant was admitted and started on intravenous acyclovir at 20 mg/kg/dose every 8 h for a total duration of 14 days. No dose adjustments were required as renal function remained normal (baseline serum creatinine 0.3 mg/dL, unchanged throughout treatment). Supportive care with gentle cleansing of ocular discharge and preservative‐free artificial tears was provided. No topical antivirals were administered initially due to concerns regarding ocular toxicity at this age. No topical corticosteroids were used at any point because there was no stromal immune‐mediated keratitis requiring anti‐inflammatory therapy. Over the two‐week course, the corneal epithelial defect resolved and stromal opacity gradually improved. The infant tolerated the antiviral therapy well without any adverse effects.

At 4 months of age, the patient presented with generalized tonic–clonic seizures and vomiting. Neurological examination showed lethargy and poor feeding. Brain magnetic resonance imaging (MRI) revealed hyperintense lesions on T2‐weighted images localized to the temporal lobes consistent with HSV encephalitis. Cerebrospinal fluid analysis demonstrated lymphocytic pleocytosis and elevated protein, with HSV DNA detected by PCR.

## 4 Conclusion and Results

The infant was managed with a second course of intravenous acyclovir for 21 days. Secondary prophylaxis (suppressive oral acyclovir) is recommended for all three forms of neonatal HSV (SEM, CNS, and disseminated/systemic disease) after completion of initial IV therapy. The standard approach is oral acyclovir suppressive therapy for 6 months. Following completion of the second course, the patient was started on oral acyclovir suppressive therapy at 300 mg/m^2^/dose three times daily for 6 months to reduce the risk of further relapse. Despite timely treatment, mild neurological deficits including delayed developmental milestones and hypotonia were noted on subsequent follow‐up examinations. Ophthalmologic evaluation documented residual corneal scarring without active keratitis. The patient was commenced on oral suppressive acyclovir to reduce the risk of further relapse. No topical antivirals or corticosteroids were ever used during the entire clinical course. The patient was followed for 2 years and is now healthy with normal development.

The neonate was admitted and started on intravenous acyclovir at 20 mg/kg every 8 h. Supportive care with gentle cleansing of ocular discharge and preservative‐free artificial tears was provided. No topical antivirals were administered initially due to concerns regarding ocular toxicity at this age. Over a 2‐week course, the corneal epithelial defect resolved and stromal opacity gradually improved. The infant tolerated the antiviral therapy well without any adverse effects.

## 5 Discussion

Neonatal HSV infections present diverse clinical challenges. Isolated ocular disease such as herpetic keratitis is rare but can be the initial or sole manifestation, necessitating high clinical suspicion for timely diagnosis [3, 4]. Isolated ocular HSV without skin or CNS involvement is extremely rare, accounting for less than 1% of all neonatal HSV cases. The presence of vesicular lesions in this case was a valuable diagnostic clue leading to PCR confirmation. The absence of maternal HSV history, common in many neonatal cases, complicates early detection [2].

PCR testing remains the gold standard for rapid and accurate diagnosis of HSV infections [1]. Intravenous acyclovir is the mainstay of treatment; however, the role of topical antivirals in neonates is less clear given potential toxicity and limited data [6]. In our case, we avoided topical antivirals entirely due to concerns about corneal epithelial toxicity in neonates, and no topical steroids were ever required.

A critical observation from this case is the risk of late CNS disease after initial SEM disease. Despite appropriate initial treatment with 14 days of intravenous acyclovir, the patient developed HSV encephalitis at 4 months of age. The literature suggests that late CNS relapse occurs in approximately 5%–10% of neonates with SEM disease, often weeks to months after the initial presentation [6]. This phenomenon underscores the importance of prolonged monitoring and consideration of suppressive antiviral therapy.

Current evidence from the AAP Red Book and the pivotal study by Kimberlin et al. supports the use of oral acyclovir suppressive therapy (300 mg/m^2^/dose three times daily for 6 months) after neonatal HSV infection to reduce the risk of neurological relapse and improve neurodevelopmental outcomes. This secondary prophylaxis is now recommended for all three disease classifications: SEM, CNS, and disseminated/systemic disease [5]. In our patient, suppressive therapy was initiated after the second hospitalization for encephalitis.

The prognosis of neonatal HSV encephalitis following treated keratitis is variable. With prompt retreatment, as in our case, mild residual deficits such as developmental delay and hypotonia are common. However, severe neurodevelopmental impairment or death occurs in up to 30% of treated cases, emphasizing the need for aggressive initial therapy and close follow‐up.

This case illustrates that even with appropriate treatment, late‐onset systemic complications including HSV encephalitis may occur, emphasizing the need for prolonged monitoring and consideration of suppressive antiviral therapy [2, 7].

Neonatal HSV infection carries a high risk of morbidity, particularly when CNS involvement develops. Early recognition and prompt antiviral treatment are fundamental to improving clinical outcomes.

This case highlights the importance of considering HSV infection in neonates presenting with ocular signs, even in the absence of systemic illness or maternal history. Early initiation of systemic antiviral therapy and vigilant long‐term follow‐up are essential to reduce morbidity and prevent severe neurological complications.

## Author Contributions

**Mohammad Saeid Sasan:** conceptualization, data curation, investigation, supervision, writing – original draft, writing – review and editing. **Amin Saeidinia:** conceptualization, data curation, writing – original draft, writing – review and editing.

## Funding

The authors have nothing to report.

## Ethics Statement

Publishing this case presentation is performed according to the ethical guidelines of Mashhad University of Medical Sciences. Patient was evaluated for her problem and her parents fulfilled informed consent for participation.

## Consent

Written informed consent for the publication of this case report and any accompanying images was obtained from the patient's legal guardian (parents). The consent form included permission to publish clinical data and imaging details in accordance with the journal's policies on patient confidentiality and privacy.

## Conflicts of Interest

The authors declare no conflicts of interest.

## Data Availability

The data that support the findings of this study are available from Akbar Hospital, Mashhad University of Medical Sciences, but restrictions apply to the availability of these data, which were used under license for the current study, and so are not publicly available. Data are however available from the authors upon reasonable request and with permission of the corresponding author.

## References

[1] D. U. De Rose , S. Bompard , C. Maddaloni , et al., “Neonatal Herpes Simplex Virus Infection: From the Maternal Infection to the Child Outcome,” Journal of Medical Virology 95, no. 8 (2023): e29024. 37592873 10.1002/jmv.29024

[2] R. J. Whitley and B. Roizman , “Herpes Simplex Viruses,” in Clinical Virology (Wiley, 2009), 409–436.

[3] M. Buzzetti , V. Silva , A. Dendi , G. Vidal , and H. Sobrero , “Neonatal Ophthalmia Caused by Herpes Simplex Virus Type I,” Andes Pediatrica 93 (2022): 749–754. 37906896 10.32641/andespediatr.v93i5.4115

[4] H. Sánchez‐Tocino , A. Villanueva , L. Giraldo , J. Anglés , and M. Pérez‐Gutiérrez , “Stromal Keratitis in a Neonate: Herpes Virus a Diagnosis to Consider,” Archivos de la Sociedad Española de Oftalmología (English Edition) 91, no. 2 (2016): 94–96. 10.1016/j.oftal.2015.10.010 26710660

[5] D. W. Kimberlin , E. Barnett , R. Lynfield , and M. H. Sawyer , Red Book 2021–2024: Report of the Committee on Infectious Diseases (American Academy of Pediatrics, 2021).

[6] A. Singh and M. Acharya , “Herpes Simplex Viral Keratitis‐A Mixed Bag of Challenging Case Scenarios,” Indian Journal of Ophthalmology‐Case Reports 3, no. 1 (2023): 58–60.

[7] H. Kimura , M. Futamura , Y. Ito , et al., “Relapse of Neonatal Herpes Simplex Virus Infection,” Archives of Disease in Childhood. Fetal and Neonatal Edition 88, no. 6 (2003): F483–F486. 14602695 10.1136/fn.88.6.F483 PMC1763242

